# Are current approaches for measuring access to clean water and sanitation inclusive of people with disabilities? Comparison of individual- and household-level access between people with and without disabilities in the Tanahun district of Nepal

**DOI:** 10.1371/journal.pone.0223557

**Published:** 2019-10-11

**Authors:** Lena Morgon Banks, Sian White, Adam Biran, Jane Wilbur, Shailes Neupane, Saurav Neupane, Aditi Sharma, Hannah Kuper

**Affiliations:** 1 International Centre for Evidence in Disability, London School of Hygiene & Tropical Medicine, London, United Kingdom; 2 Environmental Health Group, London School of Hygiene & Tropical Medicine, London, United Kingdom; 3 Valley Research Group, Kathmandu, Nepal; 4 Pennsylvania State University, State College, Pennsylvania, United States of America; Università degli Studi di Perugia, ITALY

## Abstract

**Background:**

The critical importance of safe and affordable access to water, sanitation and hygiene (WASH) is highlighted in Goal 6 of the Sustainable Development Goals, which seeks to achieve universal and equitable access for all by 2030. However, people with disabilities–who comprise 15% of the global population–frequently face difficulties meeting their WASH needs. Unmet WASH needs amongst people with disabilities may not be captured through current approaches to tracking progress towards Goal 6, which focus on household- rather than individual-level access.

**Objective:**

To evaluate access to safe water, sanitation and hygiene (WASH), at the individual- and household-level, amongst people with disabilities in the Tanahun district of Nepal.

**Methods:**

A population-based survey of disability was conducted from August-October 2016 to evaluate access to improved water and sanitation facilities between households with members with disabilities (n = 198) and those without (n = 1,265) in the Tanahun district of Nepal. A nested case-control then compared individual-level access between cases aged 15 and above with disabilities (n = 192) and age-sex-location matched controls without disabilities (n = 189), using the newly developed 21-item “Quality of WASH Access” questionnaire. Multivariate regression was used to compare household- and individual-level indicators between people and households with and without disabilities. In-depth interviews with 18 people with disabilities and their caregivers was conducted to assess the acceptability and appropriateness of the “Quality of WASH Access” questionnaire.

**Findings:**

There were no significant differences between households with and without members with disabilities in access to an improved sanitation facility or water source. However, at the individual-level, people with disabilities experienced significantly greater difficulties accessing water, sanitation and hygiene compared to people without disabilities (p<0.001 for all three scores). Amongst people with disabilities, water difficulty scores were associated with having a physical impairment and greater disability severity; sanitation difficulty scores were associated with lower socioeconomic status and physical or self-care limitations; and hygiene difficulty scores were positively associated with self-care limitations and lower socioeconomic status, and inversely associated with hearing impairments. Qualitative research found the “Quality of WASH Access” questionnaire was well understood by participants and captured many of the challenges they faced. Additional challenges not covered by the tool included: (1) time spent on WASH, (2) consistency of access, (3) sufficiency of access, and (4) dignity of access.

**Conclusion:**

People with disabilities face substantial challenges to meeting their WASH needs, particularly in using services autonomously, consistently, hygienically, with dignity and privacy, and without pain or fear of abuse. These challenges are not captured through household-level data, and so individual-level WASH access are needed to monitor progress towards universal WASH access. The Quality of WASH Access questionnaire may provide a useful data collection tool.

## Introduction

Globally, there has been impressive progress on improving access to safe water, sanitation, and hygiene (WASH) [1]. Between 1990 and 2015, 2.6 billion people gained access to an improved drinking water source and 2.1 billion people gained access to improved sanitation [1]. Still, many people experience unmet WASH needs, which can have far-reaching implications on health, food security, as well as livelihood and educational opportunities [2–5].

Access to WASH has been recognised as a fundamental human right, which is linked to the fulfilment of all other rights [6]. It has also been highlighted in Goal 6 of the Sustainable Development Goals (SDGs) as essential for global social and economic development [7]. Goal 6 emphasizes the importance of ensuring adequate and equitable access to WASH *for all*, in recognition that progress towards improving WASH has not been shared equally. For example, evidence suggests that many people with disabilities face significant challenges to accessing safe water, sanitation and hygiene on an equal basis with others [8–11]. As people with disabilities comprise upwards of 15% of the global population [12], failure to address barriers to WASH access will hamper the fulfilment of the SDG 6 and other goals, propagating the continued marginalisation of people with disabilities.

Disaggregation of data tracking progress towards SDG 6 (and all other Goals) by disability status is recommended so as “to leave no one behind” [13]. Data collection on WASH typically is collected in household surveys, where access is assumed to be consistent amongst household members. However, certain barriers to WASH access may be experienced at the individual-level. For example, people with disabilities may not be able to access the household’s facilities if they are not structurally adapted for different impairments (e.g. lack of step-free access, appropriate signage) or stigma around disability blocks use of communal facilities [8, 11, 14]. Furthermore, people with disabilities may experience lower quality of WASH access, even if they are able to use the same facilities as others. Lack of autonomy, privacy and dignity when using facilities, as well as increased pain and discomfort, have been highlighted as specific challenges for people with disabilities [8, 9]. These difficulties are rarely considered in current methods for measuring WASH inequalities, which are mainly at the household-level, or in the design of interventions to improve WASH access [15].

This study was therefore conducted to assess access to WASH at the individual- and household-level amongst people with and without disabilities, using the Tanahun district of Nepal as the study setting. Individual-level comparisons used the “Quality of WASH Access” questionnaire, which was recently developed based on qualitative research amongst people with disabilities in Malawi [8, 9]. The acceptability and appropriateness of this tool was also explored through a qualitative component.

## Methods

This study was set in Tanahun district of Nepal, with data collection running from August-October 2016. Tanahun is a predominantly rural district within Province No. 4, located in the Hills region of Nepal. In Tanahun, 67% of households have access to an improved sanitation facility and 81% have access to an improved drinking water source according to data from the 2011 Nepal Demographic and Health Survey (NDHS) [16].

Ethical approval for this research was granted by the London School of Hygiene & Tropical Medicine and the Nepal Health Research Council. Informed written consent was obtained from all study participants. A carer answered on behalf of children under 16 years old (age of consent) and people with impairments that significantly limited their ability to communicate/understand. People with disabilities with unmet health needs were referred to available services, which were mapped prior to data collection.

The study used mixed methods, including both a quantitative and qualitative component.

### Quantitative research

Quantitative data collection comprised two components: (1) a population-based household survey of disability, which compared household-level WASH access and (2) a nested case-control of people with and without disabilities to compare individual-level quality of WASH access.

#### Population-based household survey in Tanahun

A two-stage sampling strategy was employed based to select participants for the household survey [17–19]. In the first stage, 30 clusters were selected using population-proportionate-to-size sampling, with the 2011 National Census used as the sampling frame. Clusters were defined as wards within a Village Development Committee (VDC), which is the smallest administrative unit within Nepal. In the second stage, compact segment sampling was used to select households within each cluster. With this method, maps of clusters were sought in advance and then divided into segments of approximately 50 households (200 people) with assistance from local representatives. A segment was then randomly selected and all households were enumerated systematically from a random start point. Data collection stopped once the sum of household members five years and older reached 200 for that cluster.

The head of the household reported on the composition of the household (age, sex of all members) and other household-level indicators (e.g. assets, housing characteristics and facilities). Information on socioeconomic status was collected through questions on ownership of durable assets, as well as observations of housing characteristics (e.g. composition of walls, floors and roof).

All household members over five years of age were screened for disability using accepted modifications of two internationally validated question sets: the Washington Group Extended Set on Functioning for adults and the UNICEF-Washington Group Extended Set on Functioning for Children [20, 21]. Both question sets focus on an individual’s level of difficulty in performing everyday activities (e.g. seeing, walking, communicating). For most activities, the respondent has four response options: no difficulty, some difficulty, a lot of difficulty or cannot do at all. For this study, disability was defined as follows:

- Adults (≥16):
  ◦ Reported “a lot of difficulty” or “cannot do” in at least one of the following domains: seeing, hearing, walking/climbing, communicating (understanding/being understood), remembering/concentrating, self-care, upper body strength, fine dexterity.
  ◦ Reported experiencing symptoms of anxiety or depression “daily”, at a level described as “a lot”
- Children (5–15):
  ◦ Caregiver reports that compared to other children of the same age, the child experiences “a lot of difficulty” or “cannot do” in at least one of the following domains: seeing, hearing, walking, self-care, understanding, being understood, learning, remembering. Child is worried/sad “a lot more” often than other children.

Each household member reported on their own functioning, if the person was over 16 years (age of consent) and present at the time of the interview. Household heads reported on the functioning of children under 16 years, and other members not present at the time of the interview.

The remainder of the household questionnaire was answered by the household head and contained questions on the household’s water and sanitation facilities, developed based on questions in the 2011 Nepal Demographic Health Survey (DHS) [22]. Questions were included on type of facility, time to the water collection point and whether sanitation facilities were shared. The household’s water and sanitation were classified as improved if they met the following conditions, as per DHS classifications:

- Improved sanitation: flush/pour toilet, ventilated pit latrine, pit latrine with slab or composting toilet.
- Improved water source: piped water, public tap/standpipe, tube well/borehole, protected well, protected spring, rainwater, bottled water.

#### Case-control study

All people with disabilities identified in the household survey were invited to participate in the case-control study. Each case was matched to a control without a disability of similar age (+/- 5 years) and of the same sex and cluster. Controls could not be from households that included any members with disabilities, and only one control per household was permitted.

Cases and controls were interviewed using a structured questionnaire, which included the “Quality of WASH Access” questionnaire. This questionnaire was developed by three authors (HK, AB, SW), following qualitative research on disability and WASH in Malawi [8]. The tool was developed to quantify differences in WASH access at the individual-level, after observing through the qualitative research that many of the difficulties people with disabilities face in WASH access would not be captured in commonly used, household-level WASH assessments. This question set has been reviewed by international experts in disability and WASH and has also been implemented in a case-control study in Guatemala [9]. The 21-item question set focussed on the person’s experience of accessing WASH, and it contains six questions on water use, six questions on bathing, two questions on handwashing, one question on menstrual hygiene (women 15–49 only) and six questions on sanitation. Each question had yes/no response options.

The “Quality of WASH Access” module was only used for cases and controls who were 15 years of age and older to focus on adults and older children. As younger children are likely to have very different WASH needs, separate questionnaires will need to be developed to compare individual-level access in this age group. Consequently, all analyses of individual WASH access are restricted to cases and controls ages 15 and above.

#### Sample size

The required sample size for the population-based household survey was 6,000 people over five years of age, to identify adequate numbers for the case-control study (240 cases with disabilities and 240 controls, assuming 5% prevalence of disability and 80% response rate). This case-control sample size could detect an odds ratio of 1.9, assuming 80% power and a prevalence of exposure of 25% among controls.

#### Data collection procedures

Nine interviewers, divided equally into three teams, collected all quantitative data using computer tablets. Data entry forms were built using Open Data Kit (ODK), which had built in error checks. All questionnaires were translated into and administered in Nepali, and data collection was monitored by LMB for quality assurance. Data from tablets were uploaded to a secure, cloud-based server at regular intervals throughout data collection.

All interviewers underwent a five-day training prior to starting data collection. Training focused on the content and delivery of each of the questionnaires, data collection procedures (e.g. compact segment sampling, mobile data entry, case-control matching) and ethical processes (e.g. informed consent, data protection, referrals for unmet health needs).

#### Data analysis

The statistical software STATA IC 15 was used for all data analysis. Multivariate logistic regression was used to compare WASH access by disability status at either the household- or individual-level. Individual analyses were adjusted for age, sex, location (rural/urban) and socioeconomic status. Household-analyses were adjusted for the same variables, with the exception of socioeconomic status, as it was considered likely that poverty would be on the causal pathway between disability and access to improved facilities. Socioeconomic status was computed through principal component analysis (PCA) of ownership of durable household assets and livestock [23]. PCA scores were then divided into quartiles.

Access to improved water and sanitation facilities was compared between households with and without members with disabilities, using data from the population-based survey. Individual-level access to and quality of WASH was compared between people with disabilities (cases) and controls, using data from the “Quality of WASH Access” questionnaire in the case-control study. In addition to an indicator-by-indicator comparison between people with and without disabilities, three composite scores were created for water, hygiene and sanitation (S1 Table). Individual scores were divided by the maximum possible score and multiplied by 100 to obtain a score from 0–100, with higher scores indicating greater levels of difficulty. Water scores were only computed for individuals who did not have water piped into their homes.

#### Ethical considerations

Ethical approval was granted by the London School of Hygiene & Tropical Medicine and the Nepal Health Research Council. Informed written consent was received from all participants. A carer answered on behalf of children under 16 years old (age of consent) and people with impairments that significantly limited their ability to communicate/understand. People with disabilities with unmet health needs were referred to available services, which were mapped prior to data collection.

### Qualitative research on the acceptability and appropriateness of the “Quality of WASH Access” questionnaire

In-depth, semi-structured interviews were carried out with eighteen people with disabilities identified through the population-based survey, as well as through snowball sampling. Participants were purposively selected from the household survey, in order to achieve a representative sample by sex, age and impairment type. For snowball sampling, study participants and others in the community referred people with disabilities living in their area, who did not live in one of the quantitative survey’s selected segments.

Interviews centred on participants’ understanding of the questions in the tool and its acceptability. Participants were reread the questionnaire to refresh their memories, as interviews occurred one to three weeks after the quantitative survey. Interviews also discussed difficulties meeting WASH needs, in order to explore if there were domains not covered by the questionnaire. These questions on difficulties meeting WASH needs were asked before discussing the questionnaire to minimise bias. All interviews were conducted in Nepali by a researcher from Kathmandu (AS) and were recorded. Interviews were translated and transcribed from Nepali into English. Detailed notes, including observations of WASH facilities and participants’ demonstrations of WASH use, were also taken during interview visits and were used to crosscheck transcripts and provide additional contextual details. Thematic Analysis was used to analyse findings, aided by NVivo 12. Inductive, open coding of notes/transcripts was conducted (by LMB) to identify important features in the data [24]. An initial coding framework was developed based on previously conducted research and the structure of interview tool, which was then adapted iteratively based on emerging findings from the data. Codes were then collated into themes and were reviewed by a second researcher with research experience in WASH in Nepal (JW). Final themes were reviewed by in-country partners in Nepal (AS, SN, SN) and by other international experts in WASH (SW, AB).

## Results

### Sample characteristics

Overall, 6,000 household members aged over five years were enumerated across 1,469 households. Of the 5,692 people screened for disability (response rate: 94.9%), 214 individuals were identified as having a disability (prevalence: 3.8%, 95% CI: 3.3–4.3). A much larger proportion (17.2%, 95% CI: 16.2–18.2%) experienced “some difficulty” or more in at least one domain. Men were more likely to have a disability compared to women (aOR: 1.4, 1.0–1.7), after adjusting for age and location. Disability prevalence increased substantially with age (1.5% in children 5–18, 19.6% in adults over 75, p<0.001).

For the nested case-control, 418 people agreed to participate (209 cases and 209 controls, response rate 97.9%). 381 of cases and controls were 15 years and older, and thus received the WASH module. Cases and controls were well-matched by sex, location and age, as there were no significant differences in these characteristics between the two groups (Table 1). People with disabilities were more likely to belong to the poorest quartile of socioeconomic status. Physical functioning was the most commonly affected domain (50.3%) and slightly over half of respondents (53.4%) had multiple functional limitations.

**Table 1:** Description of the case-control study sample age 15+.

*Variables*	*Cases (n = 189)*	*Controls (n = 192)*	*aOR (95%CI)*
*Gender*			
Male	101 (53.4%)	107 (55.7%)	Reference
Female	85 (44.3%)	88 (46.6%)	1.1 (0.7–1.6)
*Age group*			
15–29	25 (13.2%)	29 (15.1%)	Reference
30–45	33 (17.5%)	31 (16.2%)	1.2 (0.6–2.5)
46–64	68 (36.0%)	68 (35.4%)	1.2 (0.6–2.2)
65–75 75+	30 (15.9%) 33 (17.5%)	42 (21.9%) 22 (11.5%)	0.8 (0.4–1.7) 1.7 (0.8–3.7)
*Location*			
Urban	46 (24.3%)	45 (23.4%)	Reference
Rural	147 3 (75.7%)	147 (76.6%)	0.9 (0.6–1.5)
*Socioeconomic status*			
1^st^ quartile (richest)	31 (16.4%)	44 (22.9%)	Reference
2^nd^ quartile	36 (19.1%)	47 (24.5%)	1.2 (0.6–2.2)
3^rd^ quartile	44 (23.3%)	48 (25.0%)	1.4 (0.7–2.5)
4^th^ quartile (poorest)	78 (23.3%)	53 (27.6%)	2.2 (1.2–3.9)*
*Functional domain*^*a*^			
Sensory (hearing/seeing)	68 (36.0%)	n/a	n/a
Physical (mobility, upper body, fine dexterity)	95 (50.3%)	n/a	n/a
Communication	49 (25.9%)	n/a	n/a
Cognitive (remembering, learning)	39 (20.6%)	n/a	n/a
Self-care	52 (27.5%)	n/a	n/a
Anxiety/depression	17 (9.0%)	n/a	n/a
Multiple	94 (53.4%)	n/a	n/a
*Severity score*^*b*^	0.23	0.02	<0.001

*p = <0.05 in multivariate regression;

^a^ Domains are not mutually exclusive;

^b^ Total across Washington Group domains (0 = no difficulty, 1 = some, 2 = a lot, 3 = cannot do for each domain; 3 = anxiety/depression), divided by maximum score (21 for children, 27 for adults). Scores range from 0–100%.

For the qualitative research, 18 people were recruited through purposive selection using the population-based survey data (n = 15) or through snowball sampling (n = 3) (response rate: 100%). Within this sample, ten people with disabilities were interviewed directly and for six, information was gathered by proxy. Proxies were present and assisted with communication in a further two interviews. Proxies were used for participants with severe communication/intellectual impairments, after first attempting to conduct interviews directly using available supports (sign language, simplified language). Proxies were the primary caregiver of the person with a disability, and were involved in WASH-related assistance. Participants represented an even mix by gender, and ranged in age from 15–85 years. Many participants had multiple functional limitations (n = 9), with the following breakdown reported: physical (n = 11), visual (n = 4), communication (n = 4), hearing (n = 5) and cognitive (n = 3).

### Comparing household- and individual-level WASH access

There were no significant differences in access to an improved sanitation facility or water source between households with and without members with disabilities (Table 2). Overall, over 80% of households accessed an improved, non-shared sanitation facility and over 75% had an improved source of drinking water. For most households, the water source was not on the premise, but reachable within a 30 minutes round trip.

**Table 2:** Access to water and sanitation, between households with and without members with disabilities (n = 1,463).

	Disability (n = 198)	No disability (n = 1,265)	aOR (95% CI) ^¥^
**Water**			
*Source of drinking water*			
Improved	150 (75.8%)	1,003 (79.3%)	Reference
Non-improved	48 (24.2%)	262 (20.7%)	0.8 (0.6–1.2)
*Time to obtain drinking water (round trip)*			
On premise (home/compound)	60 (30.3%)	467 (36.9%)	Reference
Less than 30 minutes	123 (62.1%)	724 (57.2%)	1.3 (0.9–1.7)
30 minutes or longer	15 (7.6%)	74 (5.9%)	1.4 (0.8–2.7)
**Sanitation**			
*Sanitation facility*			
Improved facility, not shared	165 (83.3%)	1,025 (81.0%)	Reference
Improved facility, shared	23 (11.6%)	192 (15.2%)	0.8 (0.5–1.3)
Non-improved facility	10 (5.1%)	48 (3.8%)	1.4 (0.7–2.9)

^**¥**^aOR adjusted for household size, proportion of dependents and location (rural/urban).

However, at the individual level, people with disabilities reported much greater difficulties adequately meeting their WASH needs than controls (Table 3). People with disabilities had significantly higher (worse) composite water, sanitation and hygiene difficulty scores compared to people without disabilities (p<0.001 for all scores). Furthermore, on almost all individual questions, people with disabilities fared significantly worse than people without disabilities. For example, people with disabilities were significantly more likely to need help collecting or accessing stored water, and were more likely to use a different water source compared to others in their household. Pain and fear of physical or verbal abuse when collecting water was also more common among people with disabilities compared to their peers without disabilities. Similarly, a fifth of people with disabilities required assistance when going to defecate, and were more likely to report pain, lower levels of privacy and contact with excreta when using facilities. Concerning hygiene practices, people with disabilities were more likely to use a different bathing site compared to other adults in their household, and were less likely to use a bathing place outside of their home or compound. Many people with disabilities required help for bathing or handwashing, including locating soap and other cleaning materials. Compared to people without disabilities, people with disabilities were more likely to experience pain, lower levels of privacy, contact with dirt/unsafe water and fear of abuse while bathing. Women with disabilities were also more likely to get blood on their clothes while menstruating.

**Table 3:** Assessment of WASH difficulties, between people with and without disabilities ages 15 and older (n = 381).

	Disability (n = 192)	No disability (n = 189)	aOR (95% CI)^¥^
**Water**^**Ω**^	n (%)	n (%)	
Does not collect water independently	92 (50.8%)	12 (6.4%)	18.7 (9.3–37.6)*
Uses different water source	13 (10.2%)	2 (1.1%)	11.6 (2.5–54.7)*
Experiences pain while collecting water	51 (40.2%)	4 (2.3%)	30.2 (10.4–87.9)*
Fear of abuse/violence during collection	20 (15.0%)	2 (1.1%)	15.7 (3.5–69.8)*
Cannot accessed stored water independently	47 (24.9%)	8 (4.2%)	7.9 (3.5–17.6)*
*Water score (mean*, *SE)*	*23*.*6 (1*.*4)*	*2*.*9 (0*.*6)*	*p<0*.*001*
**Sanitation**	n (%)	n (%)	
Requires help when going to defecate	39 (20.6%)	8 (4.2%)	5.8 (2.6–12.9)*
Experiences pain while going to defecate	55 (29.1%)	1 (0.5%)	81.8 (11.1–602.2)*
Contacts faeces or urine while using facility	28 (14.8%)	3 (1.6%)	10.9 (3.2–37.0)*
Has less privacy than others using site	32 (16.9%)	3 (3.1%)	6.3 (2.5–15.7)*
Afraid of abuse while going to defecate	9 (4.5%)	2 (1.0%)	4.1 (0.9–19.8)
Uses same facility as other adults in household	27 (14.3%)	10 (5.2%)	2.8 (1.3–6.1)*
*Sanitation score (mean*, *SE)*	*16*.*8 (1*.*9)*	*2*.*6 (0*.*5)*	*p<0*.*001*
**Hygiene**	n (%)	n (%)	
Bathing place outside of home, compound	74 (39.2%)	96 (50.0%)	0.6 (0.4–0.9)*
Requires help when bathing	85 (45.0%)	9 (4.7%)	20.2 (9.4–43.5)*
Uses different site than adults in household	39 (20.6%)	13 (6.8%)	3.5 (1.7–7.0)*
Afraid of abuse during bathing	18 (9.5%)	4 (2.1%)	4.8 (1.6–14.8)*
Experiences pain while bathing	71 (37.6%)	3 (1.6%)	42.1 (12.8–138.6)*
Has less privacy than others using site	61 (32.3%)	27 (14.1%)	2.8 (1.6–4.7)*
Comes into contact with dirt or dirty water	33 (17.5%)	5 (2.6%)	7.2 (2.7–19.1)*
Requires help to wash hands	49 (25.9%)	17 (8.9%)	3.6 (2.0–6.6)*
Requires help to locate cleaning materials	72 (38.3%)	4 (2.1%)	32.8 (11.4–94.2)*
Gets blood on clothing when menstruating^α^	21 (61.8%)	8 (23.5%)	5.2 (1.6–16.8)*
*Hygiene score (mean*, *SE)*	*34*.*5 (1*.*3)*	*18*.*5 (0*.*6)*	*p<0*.*001*

*p = <0.05 in multivariate regression;

^Ω^ Among people with water source outside home/compound (19 individual excluded);

^α^ Women, 15–49 only;

^**¥**^ Adjusted for age group, sex, location (rural/urban) and socioeconomic status quartile.

Amongst people with disabilities, several variables were associated with higher (worse) composite scores for difficulties in the domains of water, sanitation or hygiene (Table 4). For the water score, disability severity and having limitations in physical functioning were significantly and positively associated with greater difficulties with water access. Higher sanitation scores were significantly and positively linked to belonging to the 30–45 and 75+ year old age brackets and the poorest socioeconomic status quartile, as well as having limitations in physical and self-care domains. For hygiene, belonging to the oldest age bracket (75+) and having a limitation in the domain of self-care were positively associated with higher scores, while having difficulties in hearing was inversely associated with hygiene scores. Scores for hygiene also increased with decreasing socioeconomic status. There were no significant differences in water, hygiene or sanitation scores by gender or rural location.

**Table 4:** Regression coefficients of predictors of composite water, sanitation and hygiene scores, amongst people with disabilities (n = 189).

	Water score	Sanitation score	Hygiene score
	*Coefficient (95% CI)*	*Coefficient (95% CI)*	*Coefficient (95% CI)*
*Disability severity score*^*a*^	36.2 (0.3, 72.1)*	8.7 (-33.1, 50.4)	22.7 (-5.6, 50.9)
*Female gender*	-3.6 (-8.7, 1.5)	-3.1 (-8.5, 2.2)	0.2 (-3.8, 4.2)
*Age group*			
15–29	Reference	Reference	Reference
30–45	5.2 (-4.1, 14.5)	16.0 (5.3, 26.8)*	6.6 (-0.7, 13.9)
46–64	3.7 (-4.1, 11.8)	6.9 (-2.7, 16.5)	6.5 (-0.01, 13.0)
65–75 75+	2.6 (-7.2, 12.5) 5.2 (-5.0, 15.3)	6.4 (-5.1, 18.0) 13.2 (1.3, 25.0)*	4.8 (-3.0, 12.6) 9.1 (1.1, 17.2)*
*Rural location*	3.2 (-3.5, 9.9)	-4.3 (-11.2, 2.6)	-0.4 (-5.7, 4.9)
*Socioeconomic status*			
1^st^ quartile (richest)	Reference	Reference	Reference
2^nd^ quartile	-0.4 (-9.8, 9.1)	10.1 (-0.5, 20.6)	7.2 (0.05, 14.3)*
3^rd^ quartile	0.2 (-9.4, 9.7)	9.8 (-0.9, 20.6)	10.2 (2.9, 17.4)*
4^th^ quartile (poorest)	-0.8 (-9.9, 8.3)	11.5 (1.3, 21.6)*	9.7 (2.9, 16.6)*
*Functional domain*			
Visual	1.4 (-6.9, 9.6)	-0.6 (-10.3, 9.1)	-4.5 (-11.1, 2.1)
Hearing	-3.9 (-11.4, 3.6)	-2.9 (-11.6, 5.8)	-6.7 (-12.6, -0.9)*
Physical	14.3 (6.7, 21.9)*	11.5 (2.7, 20.4)*	5.3 (-0.7, 11.3)
Communication	-4.6 (-12.1, 2.9)	6.2 (-2.6, 14.9)	-1.6 (-7.5, 4.3)
Cognitive	-1.7 (-9.7, 6.3)	6.4 (-3.0, 15.8)	4.0 (-2.4, 10.4)
Self-care	6.7 (-0.8, 14.3)	29.9 (20.9, 38.8)*	15.3 (9.3, 21.4)*
Anxiety/depression	8.9 (-1.6, 19.4)	4.3 (-8.0, 16.5)	0.2 (-8.1, 8.4)

*p = <0.05 in multivariate regression;

^a^ Total across Washington Group domains (0 = no difficulty, 1 = some, 2 = a lot, 3 = cannot do for each domain; 3 = anxiety/depression), divided by maximum score (21 for children, 27 for adults). Scores range from 0–100%.

### Acceptability and validity of the “Quality of WASH Access” questionnaire amongst people with disabilities

All participants in the qualitative research reported that the questions in the “Quality of WASH Access” tool were easily understood and captured the majority of challenges they faced in meeting their WASH needs. The appropriateness of the WASH tool domains was further reinforced as many of the challenges–particularly experiences of pain and need of assistance–were reiterated during in-depth interviews or observed during WASH demonstrations. Observations of participants’ WASH facilities and their use of them also highlighted persistent accessibility challenges: difficult terrain, long distances and lack of modifications at point of use (e.g. steps, no rails). Further, observations revealed that participants may underestimate WASH difficulties. For example, a few participants reported in the quantitative survey–and qualitative interviews–that they did not come into contact with urine or faeces when using toilet facilities; however when demonstrating how they would typically access their toilet facility, these participants had to place their hands on the unclean floors surrounding the squat toilet in order to balance.

A few additional factors affecting quality of WASH access were also raised, specifically: (1) time spent on WASH activities, (2) consistency of access, (3) sufficiency of access; and (4) dignity of access.

#### Time spent on WASH activities

Many people with disabilities and their caregivers reported that they spent more time on WASH-related activities in comparison to other people in their household or their community. For example, getting to facilities could take longer, as could time spent on tasks such as bathing and washing clothes. As an illustration, a 50-year-old woman with a physical impairment explained that while others could wash their clothes in “two hours”, it took her “a whole day”.

Furthermore, some people with disabilities had additional WASH needs, resulting in further time outlays. In particular, caregivers of people with incontinence reported spending substantial amounts of time on frequent bathing and washing of clothes, bedding and other items–often multiple times per day.

#### Consistency of access

Inconsistent WASH access was mentioned in interviews as an issue affecting people with disabilities, as well as others in their household or community. For example, almost half of respondents explained that they did not consistently use the same water source throughout the year (including households with piped water on the premise) as their preferred water source ran out at times due to seasonality, overuse or pipe breakages. Although these challenges could affect everyone relying on the same source, people with disabilities appear particularly affected. For example, when alternative sources were further away or involved more challenging terrain, people with disabilities in some instances were either not able to access the source or experienced greater levels of difficulty in doing so.

In addition to shared challenges, interviews also highlighted disability-specific difficulties to consistently accessing WASH. Notably, as many people with disabilities required assistance in meeting their WASH needs, access could depend on caregivers’ availability or willingness to provide assistance. For example, a 63-year-old man with a physical impairment explained that he would often go weeks without bathing as his wife “…is also busy….I only take a bath when she’s free.” Fluctuations in level of functioning could also lead to variations in WASH access.

#### Sufficiency of WASH access

Interviews indicated that people with disabilities faced challenges in sufficiently meeting their WASH needs. In particular, many reported bathing less frequently or limiting water intake compared to other household members. For example, a man with a profound visual impairment explained he felt “uneasy bathing inside the bathroom because I can’t see. So I take a bath once every 10–15 days or once every month. Last time I didn’t take a bath for 7 months.” Unmet WASH needs were often linked to the need for assistance, difficulties getting to facilities and increased levels of pain, amongst other reasons.

#### Dignity of access

Closely linked to the lack of autonomy and privacy, some interviews highlighted that accessing WASH with dignity was a challenge. Several respondents reported feeling uncomfortable or demoralised that they required assistance to complete personal activities such as bathing or using the toilet. Additionally, lack of adaptations to facilities or availability of support could result in both unhygienic and undignified use. For example, the caregiver of a 17-year-old girl, who had intellectual and communication impairments and who was incontinent, reported that her daughter would sit in a plastic chair in her urine or faeces for hours at a time if she was unable to leave her work in the fields to assist her with going to the toilet.

## Discussion

The SDGs advance a mandate to “leave no one behind” in all development efforts, which is reflected in Goal 6 on universal WASH access [7]. However, this research highlights that people with disabilities face substantial challenges to meeting their WASH needs, particularly in using facilities independently, hygienically, and without pain or fear of abuse. It is important to consider these difficulties when measuring progress towards SDG 6 and other initiatives, as this research highlights that quality of access can differ substantially between people with and without disabilities even when household-level availability of improved water and sanitation facilities is good.

Due to the link between disability and poverty [25], it was expected that households with members with disabilities might experience decreased access to improved water and sanitation facilities. However, no differences were observed between households with and without members with disabilities. This finding mirrors other research from Cameroon, India, Malawi and Bangladesh [10], as well as from other areas in Nepal [26]. Research in Guatemala even found households with members with disabilities were more likely to use an improved sanitation facilities [9]. The lack of difference between households with and without members with disabilities in Tanahun may reflect universally high access to improved water and sanitation facilities in the study sample (>75% for improved water, >80% for improved sanitation in this study), and as reported in other areas of Nepal [27].

In contrast, there were large disparities at the individual-level between people with and without disabilities using the newly developed “Quality of WASH Access” questionnaire. People with disabilities fared significantly worse on almost all indicators in comparison to people without disabilities. A case-control study in eastern Guatemala using this tool found similar disparities in accessing sanitation and hygiene; however there were no significant differences in water access scores [9]. The latter finding contrasts with this research, as the gap between people with and without disabilities in Tanahun for the three WASH scores was highest for reported water access difficulties. This difference is likely explained by distance of the water source, as the vast majority (90%) of households in Guatemala used a source on their compound, compared to 30% in Tanahun.

This research also highlights that difficulties in WASH access are not homogenous amongst people with disabilities. Overall, people with physical impairments and self-care limitations experienced the greatest challenges adequately meeting their WASH needs. Although not explicitly captured in the quantitative component, the qualitative research in this and other studies have highlighted that some of these challenges may be linked to incontinence or difficulties managing the difficult terrain in this region of Nepal [8, 16]. Additionally, older age amongst people with disabilities was linked to greater sanitation and hygiene difficulties, which was also found in the study from Guatemala [9]. Furthermore, people with disabilities in the poorest socioeconomic quartile experienced the highest water and sanitation difficulty scores. Poverty may be linked to poorer WASH access, due to inability to afford or lack of information on adaptations that could improve ease of use, as well as autonomous and hygienic access to facilities. Alternatively, lower socioeconomic status may increase vulnerability to disability, and resulting lower levels of functioning could hinder WASH access [28–30]. Finally, other research has found additional predictors of unmet WASH needs amongst people with disabilities, such as living in a rural area [8, 9]. However, as the majority of the study population was rural-based, this study may not have been adequately powered to detect rural-urban differences. Similarly, compared to men, women with disabilities often have additional WASH needs related to menstrual hygiene management, as well as an increased expectation to participate in WASH activities, which was not a risk factor in the present study.

The inequalities at the individual-level highlight the need for additional data on intra-household access to and quality of WASH, particularly covering domains that are of importance for people with disabilities. Most indicators of WASH access–including in the monitoring of SDG 6 –are measured at the household-level, which likely masks difficulties faced by people with disabilities in meeting their WASH needs [7, 31]. The “Quality of WASH Access” questionnaire may be a useful tool for better understanding individual-level challenges to adequately meeting WASH needs. Qualitative research revealed the “Quality of WASH Access” tool was well understood by participants and captured the majority of the challenges people with disabilities face in meeting their WASH needs.

Some additional questions not covered in the tool may help capture other difficulties faced by people with disabilities. First, questions on time spent on WASH-related activities is important for gauging access, and could also be used explore the opportunity costs of non-inclusive WASH. Second, questions on the consistency of access to facilities is a concern for both people with and without disabilities. Some routine indicators already capture issues around intermittent access. For example, “safely managed” drinking water and sanitation services, as classified in indicators 6.1.1 and 6.1.2 of SDG 6, are defined as “being available when needed”[32]. However, data collected for these indicators are still typically measured at the household-level, while people with disabilities may face greater inconsistencies in access due to reliance on others or greater sensitivity to changes in climate or terrain. Third, indicators on sufficiency of WASH access would be helpful for identifying unmet WASH needs. For instance, comparing bathing frequency or water use to other people in the household or community could capture inequalities in access. Finally, it is important to explore whether individuals can meet their WASH needs in a way that is dignified and acceptable to them. Adding questions on these topics to the “Quality of WASH Access” tool could improve its utility in capturing the range of WASH difficulties experienced by people with disabilities, which have been included in an updated version of the questionnaire (S1 File). These new questions will require pilot testing and should be used in conjunction with household-level measures on safely managed water and sanitation [31]. Further, additional questionnaires targeted to caregivers may be needed to explore the impact of WASH access challenges on households with members with disabilities, such as time and costs associated with WASH-related caregiving.

Overall, continued measurement of individual-level WASH access is needed to ensure people with disabilities are being included in progress towards universal access to WASH. Additionally, research trialling and measuring the effectiveness of interventions could provide solutions to improve access to and quality of WASH for people with disabilities. For example, some programmes have noted that low cost interventions, which utilise locally available material, can improve the accessibility of facilities (e.g. placing a chair with a hole cut in the seat over a pit latrine for a person who finds squatting difficult, attaching wooden handrails to latrine walls) [33]. Providing information about different options allows households to adapt their facilities themselves according to their need and budget [34, 35]. Further, involving people with disabilities in community events on WASH, such as Community-Led Total Sanitation, can help ensure planned activities are inclusive and meet the needs of people with disabilities [15, 35].

### Strengths and limitations

Some limitations should be taken into account when interpreting the results of this research. This study was set in the Tanahun district of Nepal, which is predominantly rural and was identified by stakeholders as having better availability of disability-support services than other areas of Nepal. Consequently, findings may not be generalizable to the rest of the country. In addition, as the individual WASH questionnaire was limited to people ages 15 and older, more research is needed to look at WASH access issues in younger children. Finally, the “Quality of WASH Access” questionnaire has not undergone psychometric testing and member checking of qualitative findings were not undertaken.

A major strength of this study was the use of mixed methods. This approach allowed for a broad assessment of WASH access at both the individual- and household-level, with qualitative research to triangulate findings across methodologies and instruments, which strengthens the validity of the results. Further, all the participants in the quantitative and the majority of participants in the qualitative were selected through population-based recruitment, which improves the generalisability of the study’s findings.

## Conclusion

People with disabilities faced significant difficulties meeting their WASH needs, with many experiencing increased pain and time allocation, as well as reduced privacy, dignity and autonomy compared to people without disabilities. Current measures of WASH access fail to capture challenges faced by people with disabilities, which carries implications for monitoring progress towards achieving universal WASH access for all. The “Quality of WASH Access” questionnaire is a novel instrument, which could help better understand individual-level WASH access difficulties. Measuring and addressing disparities in individual-level access is essential for fulfilling the SDG’s mandate to “leave no one behind”.

## Supporting information

S1 Table Items contributing towards the water, sanitation and hygiene scores. (DOCX)

S1 File Updated quality of WASH access questionnaire. (DOCX)
