# Supplementary material for: Are current approaches for measuring access to clean water and sanitation inclusive of people with disabilities? Comparison of individual- and household-level access between people with and without disabilities in the Tanahun district of Nepal
Source: PLoS One. 2019 Oct 11;14(10):e0223557. doi: 10.1371/journal.pone.0223557 (PMC6788693; doi:10.1371/journal.pone.0223557)
Supplement: S1 File — (DOCX) [file pone.0223557.s002.docx]

Quality of WASH access Questionnaire

**Information for enumerator:**

This questionnaire is to be administered to any person who is over the age of 15. This questionnaire is designed to be used with people who may have particular challenges accessing WASH, such as people with disabilities.

The sample questions are designed to assess whether participants can independently answer questions. If the individual is unable to answer the sample questions by themselves (e.g. unable to communicate due to a hearing or cognitive impairment) then the questions can be asked to their caregiver. In order to do this questions must be re-phrased. For example instead of asking ‘Do you ever go and collect water?’ you would say ‘Does ______ (name of disabled family member) ever go and collect water?’. Responses from caregivers should be analysed and presented separately.

In general, this questionnaire should be conducted in a private location. In cases where a disabled participant has communication challenges a family member may be asked to aid communication (e.g. through sign language interpretation).

**Explain to the participant:**

I would like to ask you some questions about your water use, your bathing, your handwashing and your defecation practices. The questions refer to what you do most of the time when you are at home. I want to begin with some questions about you.

**Sample questions**

1. What is your age?

**Yes No**

1. Do you live in this house?
2. Have you experienced any pain today?

**Mark the following:**

This questionnaire was conducted with the primary participant

This questionnaire was conducted with the caregiver of the primary participant due to an inability to answer the sample questions

**Explain to the participant:**

Now I am going to ask you some questions about your water use.

1. **Water Use**
   1. Who mainly collects water in your community? (read option – more than one can be ticked if appropriate)
2. Men
3. Women
   1. In your community what age are the people who normally collect water? (read options - more than one can be ticked if appropriate)
   2. Under 15 years of age
   3. 15 to 50 years of age
   4. Over 50 years of age

**Yes No**

- 1. Do you ever go to collect water?
  2. (If yes to 1c): When you go to collect water, do you need help from others?
  3. (If yes to 1c): Do you collect water from the same source(s) as other members of your household?
  4. Do you take the same amount of time to collect water compared to other household members?
  5. (If yes to 1c): Can you collect the same quantities of water as other members of your household?
  6. (If yes to 1c): Does collecting water cause you additional pain?
  7. (If yes to 1c): Are you afraid of physical or verbal abuse or violence when you collect water?
  8. Can you access stored water in your home without help from others?
  9. Are you able to access enough water to meet your daily needs?

**Explain to the participant:**

Now I am going to ask you some questions about your bathing practices.

1. **Bathing**

**Yes No**

- 1. Do you usually need help from others when you go for bathing?
  2. (If yes to 2a): Do you always get this help when you need it?
  3. (If yes to 2a): Do you feel embarrassed or disrespected when

receiving this help?

- 1. When you are at home where do you usually bathe? (read options)

In the house, compound or yard

At a tap or pump away from the compound

At a surface water source away from the compound (stream/river/pond/lake/sea etc).

- 1. How often do you usually bathe?
     1. Every day
     2. Two or more days per week
     3. Once a week
     4. Two or more times per month
     5. Once a month or less
  2. Do you use the same place for bathing as other adult members of your household?
  3. Does it cause you additional pain to use this place?
  4. Do you use this place with as much privacy as other people?
  5. Are you afraid of physical or verbal abuse or violence when you use this place for bathing?
  6. Do you use this place without coming into contact with dirt or dirty water?

**Explain to the participant:**

Now I am going to ask you some questions about handwashing.

1. **Handwashing Yes No**
2. Are you able to wash your hands with soap and water without help from others?
3. Are you able to locate and use soap or other cleansing materials without help from others?

**Explain to Female Participants:**

Now I am going to ask you about your experiences while menstruating.

1. **Menstruation Yes No**
   1. When you are menstruating do you often get blood on your clothing?

**Explain to the participant:**

Now I am going to ask you some questions about your defecation practices.

1. **Sanitation Yes No**
   1. Do you usually need help from others when you go for defecation?
   2. (If yes to 5a): Do you always get this help when you need it?
   3. (If yes to 5a): Do you feel embarrassed or disrespected when

receiving this help?

- 1. When you are at home where do you usually go for defecation? (read options)

1. In the bush, field or somewhere around the house
2. In a chamber pot / bucket at the house
3. Toilet or latrine owned by this household
4. Toilet or latrine owned by a neighbouring household
5. Public toilet
   1. Does it cause you additional pain to use this place?
   2. Is this the same place that other adults in your household usually use for defecation?

- 1. Do you use this place without coming into contact with faeces or urine?
  2. When you go for defecation do you have as much privacy as other members of your household?
  3. Are you afraid of physical or verbal abuse or violence when you go for defecation?
